# Substantial blood loss and transfusion burden after periacetabular osteotomy: Benchmark estimates from a systematic review and meta‐analysis

**DOI:** 10.1002/ksa.70432

**Published:** 2026-06-03

**Authors:** Nikolai Ramadanov, Jonathan Lettner, Roland Becker, Robert Prill, Marko Ostojic, Sufian S. Ahmad

**Affiliations:** ^1^ Center of Orthopaedics and Traumatology, Brandenburg Medical School University Hospital Brandenburg an der Havel Brandenburg an der Havel Germany; ^2^ Faculty of Health Science Brandenburg Brandenburg Medical School Theodor Fontane Brandenburg an der Havel Germany; ^3^ Sports Traumatology Division, Traumatology Department “Draškovićeva” University Hospital “Sisters of Mercy” Zagreb Croatia; ^4^ Osteon Orthopedics and Sports Medicine Clinic Mostar Bosnia and Herzegovina; ^5^ Department of Orthopaedic Surgery Hannover Medical School Hannover Germany

**Keywords:** blood loss, blood transfusion, hip dysplasia, meta‐analysis, periacetabular osteotomy, perioperative management

## Abstract

**Purpose:**

To establish pooled benchmark estimates for perioperative blood loss and transfusion burden after periacetabular osteotomy (PAO) and to explore potential study‐level moderators across the available literature.

**Methods:**

A systematic search of PubMed, Embase, Epistemonikos and CENTRAL was performed up to 30 March 2026. Prospective and retrospective primary clinical studies reporting at least one predefined perioperative blood management outcome after PAO were included. Outcomes comprised transfusion rate, units transfused, intraoperative blood loss, estimated blood loss, haemoglobin drop and postoperative haemoglobin levels. Random‐effects meta‐analyses of proportions and means were performed. Univariable meta‐regression was used to explore study‐level moderators.

**Results:**

Forty‐seven studies comprising 4402 patients and 4767 hips were included. The pooled transfusion rate was 16.2% (95% confidence interval [CI] = 11.1–23.0), and the pooled mean number of transfused units was 1.94 (95% CI = 1.32–2.55). The pooled mean intraoperative blood loss was 961.8 mL (95% CI = 804.1–1119.5), and the pooled mean estimated blood loss was 809.9 mL (95% CI = 706.4–913.5). The pooled mean haemoglobin drop was 32.4 g/L (95% CI = 27.7–37.1), and the pooled mean postoperative haemoglobin level was 99.4 g/L (95% CI = 97.2–101.6). Longer operative time was significantly associated with higher intraoperative and estimated blood loss. Higher preoperative haemoglobin was associated with greater haemoglobin drop, while higher age was associated with transfusion‐related outcomes.

**Conclusion:**

PAO is associated with substantial perioperative blood loss and a relevant transfusion burden. These pooled estimates provide clinically useful benchmark values for perioperative blood management after PAO.

**Level of Evidence:**

Level III systematic review and meta‐analysis of predominantly retrospective cohort studies.

**Abbreviations**
BDDH: borderline developmental dysplasia of the hip
BMI: body mass index
CENTRAL: Cochrane Central Register of Controlled Trials
CI: confidence interval
CRD: Centre for Reviews and Dissemination/PROSPERO registration identifier prefix
DDH: developmental dysplasia of the hip
Hb: haemoglobin
*I* ^2^
: Higgins heterogeneity statistic
PAO: periacetabular osteotomy
PRISMA: Preferred Reporting Items for Systematic Reviews and Meta‐Analyses
PROSPERO: International Prospective Register of Systematic Reviews
RCT: randomized controlled trial
REML: restricted maximum likelihood
ROBINS‐I: Risk Of Bias In Non‐randomized Studies of Interventions
RoB 2: Risk of Bias 2
SD: standard deviation
THA: total hip arthroplasty
TXA: tranexamic acid

## INTRODUCTION

Periacetabular osteotomy (PAO) is an established joint‐preserving procedure for the treatment of symptomatic acetabular dysplasia and has demonstrated favourable mid‐ to long‐term outcomes in appropriately selected patients [94]. Despite these encouraging results, PAO remains technically demanding and is consistently associated with substantial perioperative blood loss and a relevant rate of blood transfusion. Accordingly, perioperative blood management represents a key component of modern PAO care.

Several studies have evaluated blood management strategies in PAO, with particular focus on antifibrinolytic agents. Available evidence indicates that tranexamic acid (TXA) reduces perioperative blood loss and, in part, transfusion requirements without increasing complication rates [101, 110]. However, prior work has predominantly focused on specific interventions rather than on the overall perioperative blood loss profile associated with PAO.

In addition, the PAO literature is characterized by considerable heterogeneity in surgical technique, patient selection and perioperative management, all of which may substantially influence blood loss and transfusion‐related outcomes [20]. This variability complicates interpretation across studies. In total hip arthroplasty (THA), perioperative blood loss and transfusion burden are influenced by patient‐ and procedure‐related factors, including preoperative haemoglobin, operative time and patient characteristics [15, 71]. Whether similar patterns apply to PAO remains unclear. Importantly, pooled benchmark estimates for key perioperative blood management outcomes after PAO are currently lacking. Such data are clinically relevant, as they may provide a reference framework for perioperative decision‐making, patient counselling and interpretation of interventional studies.

Therefore, the aim of the present systematic review and quantitative synthesis was to establish pooled benchmark estimates for perioperative blood loss and transfusion burden after PAO and to explore potential study‐level moderators using meta‐regression. We hypothesized that PAO is associated with substantial perioperative blood loss and a relevant transfusion burden, and that study‐level factors such as operative time, preoperative haemoglobin and patient characteristics are associated with variability in these outcomes.

## METHODS

### Protocol registration and reporting standards

This systematic review was prospectively registered in the International Prospective Register of Systematic Reviews (PROSPERO) on 18 February 2026 (CRD420261309388). The review was conducted in accordance with the Preferred Reporting Items for Systematic Reviews and Meta‐Analyses (PRISMA) 2020 statement [69]. The completed PRISMA checklist is provided in Table S1.

### Search strategy

A comprehensive literature search was performed in four electronic databases: PubMed, Embase, Epistemonikos and the Cochrane Central Register of Controlled Trials (CENTRAL). The final search was conducted on 30 March 2026. The search strategy combined controlled vocabulary and free‐text terms related to PAO and hip dysplasia using the following Boolean structure: ((periacetabular osteotomy OR PAO OR Bernese) AND (dysplasia OR DDH OR borderline dysplasia OR BDDH)). Search syntax was adapted as required for each database. No restrictions on publication date were applied.

### Study screening and eligibility

Two reviewers (N.R. and J.L.) independently screened all records in a two‐step process: (1) title and abstract screening and (2) full‐text assessment. Disagreements were resolved through discussion until consensus was reached. Inter‐reviewer agreement was quantified using Cohen's kappa (*κ*).

Eligible studies included prospective or retrospective primary clinical studies investigating PAO and reporting at least one perioperative blood management‐related outcome. Both comparative studies and single‐arm cohorts were considered eligible. Studies were excluded if they were reviews, editorials, case reports, animal studies or grey literature.

### Blood management outcomes

The predefined blood management outcomes were grouped into three domains: (i) transfusion outcomes, including transfusion rate and units transfused; (ii) blood loss parameters, including intraoperative blood loss and estimated blood loss; and (iii) haemoglobin‐related outcomes, including haemoglobin drop and postoperative haemoglobin levels. These outcomes were selected because they represent the most consistently reported and clinically relevant perioperative markers of blood loss and transfusion burden after PAO.

### Potential moderators

To explore sources of between‐study variability, prespecified study‐level moderators were grouped into three categories: (i) primary moderators, including TXA use, cell saver use, autologous predonation, preoperative haemoglobin and operative time; (ii) secondary moderators, including mean age, body mass index (BMI) and female sex; and (iii) exploratory moderators, including length of stay, complications, reoperations and conversion to THA. These variables were selected based on their potential relevance to perioperative blood loss, transfusion requirements or overall surgical complexity.

### Data extraction

Data extraction was performed independently by two reviewers (N.R. and J.L.) using a standardized, predefined data collection form. Extracted variables included study characteristics (design, sample size), patient demographics, perioperative parameters and follow‐up duration. Inclusion in the quantitative synthesis required explicit reporting of at least one predefined blood management outcome.

For missing data, a predefined hierarchical approach was applied: (1) contacting corresponding authors; (2) estimating standard deviations (SDs) from reported ranges using the formula (maximum − minimum)/4 [103]; and (3) imputing missing SDs where necessary using established methods [8]. For studies reporting multiple treatment arms unrelated to the predefined blood management interventions of interest, the respective arms were combined into a single pooled cohort to avoid double‐counting and to permit inclusion in the overall arm‐based synthesis.

### Quality assessment

Risk of bias was assessed independently by both reviewers using the Risk Of Bias In Non‐randomized Studies of Interventions (ROBINS‐I) tool for non‐randomized studies [89] and the Risk of Bias 2 (RoB 2) tool for randomized controlled trials [90]. Disagreements were resolved through consensus.

### Statistical analysis

All statistical analyses were performed using R (R Foundation for Statistical Computing). The primary aim was to derive pooled benchmark estimates for perioperative blood management outcomes following PAO.

For continuous outcomes, pooled means with 95% confidence intervals (CIs) were calculated using random‐effects meta‐analysis of single‐arm data. For transfusion rate, pooled proportions with 95% CIs were estimated using a random‐effects model. Between‐study heterogeneity was assessed using the Higgins *I* ^2^ statistic, with values of <25%, 25%–75% and >75% indicating low, moderate and high heterogeneity, respectively. Random‐effects models were used throughout because of expected clinical and methodological heterogeneity.

To investigate study‐level factors associated with the pooled outcomes, univariable meta‐regression analyses were performed using the prespecified moderators. Continuous variables were analyzed using reported study‐level means, whereas binary variables such as TXA use, cell saver use and autologous predonation were coded dichotomously. Count variables were converted to proportions where appropriate. Meta‐regression results are reported as regression coefficients (*β*) with 95% CIs and corresponding *p* values and were considered exploratory. A two‐sided *p* value < 0.05 was considered statistically significant.

## RESULTS

### Search results

**Figure 1:**
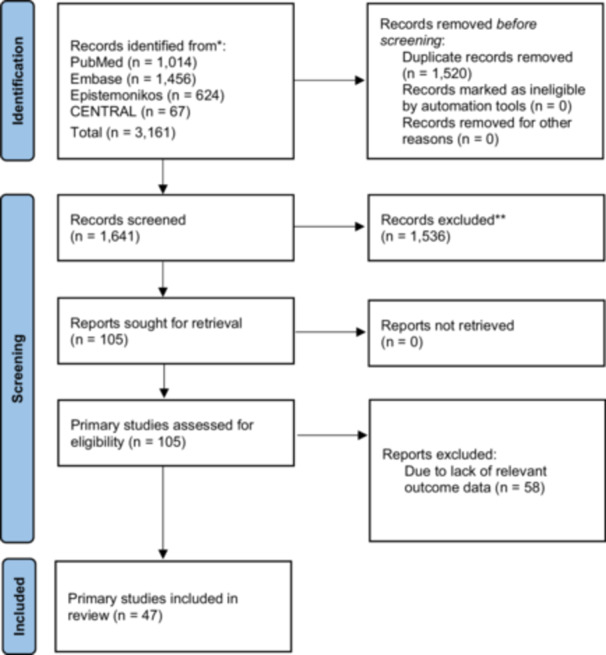
PRISMA 2020 flow diagram illustrating study selection process. CENTRAL, Cochrane Central Register of Controlled Trials; PRISMA, Preferred Reporting Items for Systematic Reviews and Meta‐Analyses.

A total of 1014 records were identified through PubMed, 1456 through Embase, 624 through Epistemonikos and 67 through the CENTRAL. After removal of 1520 duplicates, 1641 records remained for title and abstract screening. Screening was performed independently by two reviewers with excellent inter‐reviewer agreement (*κ* = 0.98). A total of 105 full‐text articles [1, 2, 3, 4, 5, 6, 7, 8, 9, 10, 11, 12, 13, 14, 16, 17, 18, 19, 21, 22, 23, 24, 25, 26, 27, 28, 29, 30, 31, 32, 33, 34, 35, 36, 37, 38, 39, 40, 41, 42, 43, 44, 45, 46, 47, 48, 49, 50, 51, 52, 53, 54, 55, 56, 57, 58, 59, 60, 61, 62, 63, 64, 65, 66, 67, 68, 70, 72, 73, 74, 75, 76, 78, 79, 80, 81, 82, 83, 84, 85, 86, 87, 88, 91, 92, 93, 95, 96, 97, 98, 99, 100, 102, 104, 105, 106, 107, 108, 109, 111, 112, 113, 114, 115] were assessed for eligibility, with complete agreement between reviewers (*κ* = 1.0). Of these, 58 studies [1, 4, 5, 6, 9, 10, 12, 17, 18, 19, 21, 22, 23, 25, 26, 27, 28, 29, 30, 31, 34, 35, 36, 37, 38, 41, 42, 43, 45, 48, 49, 50, 59, 60, 62, 63, 64, 65, 66, 67, 68, 70, 76, 78, 79, 80, 81, 91, 92, 93, 97, 104, 105, 107, 108, 109, 115] were excluded due to lack of relevant outcome data. Ultimately, 47 primary studies [2, 3, 7, 8, 11, 13, 14, 16, 24, 32, 33, 39, 40, 44, 46, 47, 51, 52, 53, 54, 55, 56, 57, 58, 61, 72, 73, 74, 75, 82, 83, 84, 85, 86, 87, 88, 95, 96, 98, 99, 100, 102, 106, 111, 112, 113, 114] were included in the final meta‐analysis (Figure 1).

### Study and patient characteristics

A total of 47 studies [2, 3, 7, 8, 11, 13, 14, 16, 24, 32, 33, 39, 40, 44, 46, 47, 51, 52, 53, 54, 55, 56, 57, 58, 61, 72, 73, 74, 75, 82, 83, 84, 85, 86, 87, 88, 95, 96, 98, 99, 100, 102, 106, 111, 112, 113, 114] were included and were predominantly retrospective in design, with most representing Level III or IV evidence. Only a small number of higher‐level studies were identified, including two prospective cohort studies [39, 44] and two randomized comparative studies [52, 56]. The studies were published between 1999 and 2025 and originated from multiple geographic regions, including North America, Europe and Asia (Table 1).

**Table 1:** Study characteristics and patient demographics of included studies.

Author	Year	Country	Journal	Study design	Level of evidence	Study period	Follow‐up (years ± SD; range)	Technique	Surgical approach	Concomitant procedures	Patients/hips (*N*)	Female sex (*N*)	Mean age (year ± SD; range)	BMI (kg/m^2^ ±SD; range)	Remarks
Albers CE [2]	2013	Switzerland	*Clinical Orthopaedics and Related Research*	Retrospective cohort study	3	1984–1987; 1997–2000	11.0 ± 1.0; 10.0–14.0	Bernese	Modified Smith–Petersen	Osteochondroplasty (57%), Labrum procedures (49%), intertrochanteric osteotomy (10%–23%)	147/165	94	29.0	23.0 ± 4.0; 19.0–33.0	NR
Amano T [3]	2014	Japan	*Hip International*	Retrospective cohort study	3	1989–2007	13.3; 5.0–21.0	Eccentric rotational acetabular osteotomy	NR	NR	108/108	72	35.9	21.8 ± 2.2	NR
Atwal NS [7]	2008	UK	*Hip International*	Retrospective cohort study	3	1996–2005	NR	Bernese	Modified Stoppa approach	NR	107/122	109	23.6; 18.7–28.6	NR	NR
Baraka MM [8]	2022	Egypt	*SICOT‐J*	Prospective case series	4	2016–2018	3.2; 2.0–5.0	Bernese	Modified Stoppa approach	NR	9/9	7	22.4; 15.0–30.0	NR	NR
Bernstein P [11]	2007	Germany	*The Open Orthopaedics Journal*	Retrospective comparative study	3	NR	1.6; 0.5–2.4	Bernese	Modified Smith–Petersen	Labrum/FAI: 2; Femoral osteotomy: 4	23/23	15	27.0; 16.0–40.0	NR	NR
Bryan AJ [13]	2016	USA	*Orthopaedics*	Retrospective cohort	3	2003–2014	1.3–5.8	Bernese	Modified Smith–Petersen	NR	137/150	115	26.4 ± 8.7	26.0 ± 5.3	NR
								Bernese	Modified Smith–Petersen	NR	68/75	58	24.8 ± 8.2	26.0 ± 6.3	TXA
								Bernese	Modified Smith–Petersen	NR	69/75	57	28.0 ± 9.2	26.0 ± 4.3	Non‐TXA
Burke NG [14]	2011	Ireland	*Acta Orthopaedica Belgica*	Retrospective case series	4	1998–2005	4.9; 1.3–8.0	Bernese	Modified Smith–Petersen	NR	79/85	72	22.9; 14.0–41.0	NR	NR
Clohisy JC [16]	2005	USA	*Journal of Bone and Joint Surgery. American Volume*	Retrospective case series	4	1994–2001	4.2; 1.9–8.1	Bernese	Modified Smith–Petersen	6 proximal femoral osteotomies	13/16	1	17.6; 13.0–31.8	NR	NR
Domb BG [24]	2015	USA	*Arthroscopy: The Journal of Arthroscopic and Related Surgery*	Retrospective case series	4	2010–2013	2.4; 0.6–3.3	NR	Modified Smith–Petersen	NR	17/17	14	24.2 ± 7.1	24.3 ± 4.8	NR
Guo H [32]	2024	China	*Orthopaedic Surgery*	Retrospective comparative study	3	2018–2022	NR	Bernese	Modified Smith–Petersen	NR	56/62	48	27.6 ± 7.3	21.2 ± 1.9	NR
Haertlé M [33]	2024	Germany	*Bone & Joint Journal*	Retrospective case series	4	2022–2023	NR	Bernese	NR	Offset correction: 27, Femoral osteotomy: 3, Implant removal: 3, Surgical dislocation: 1	106/118	90	23.4 ± 7.5	24.9 ± 4.6; 17.3–37.8	NR
Khan OH [39]	2017	UK	*Bone & Joint Journal*	Prospective cohort study	2	2010–2013	2.8; 1.2–4.5	Bernese	Modified Smith–Petersen	NR	151/166	136	32.0; 15.0–56.0	NR	NR
Kim HT [40]	2009	South Korea	*Journal of Bone and Joint Surgery. British Volume*	Retrospective comparative study	3	2001–2008	NR	Bernese	Mixed (single anterior + dual AP)	NR	23/26	19	19.4; 11.3–38.0	NR	NR
Kraeutler MJ [44]	2018	USA	*Journal of Hip Preservation Surgery*	Prospective cohort study	2	NR	2.0	Bernese	NR	Staged hip arthroscopy (all patients)	124/145	118	30.1 ± 8.9; 15.0–50.0	23.8 ± 5.1; 16.3–44.9	NR
Lee CB [46]	2013	USA	*Hip International*	Retrospective cohort study	3	2009–2011	NR	Bernese	Abductor‐sparing	NR	141/169	125	25.6 ± 9.7	25.2 ± 4.8	NR
Lee SH [47]	2025	USA	*International Journal of Computer Assisted Radiology and Surgery*	Prospective case series	4	NR	1.2	Bernese	Navigation	HAS: 1	5/5	4	25.8 ± 9.1	24.3 ± 3.1	
Lerch TD [51]	2017	Switzerland	*Clinical Orthopaedics and Related Research*	Retrospective cohort study	3	1984–1987	29.0; 27.0–32.0	Bernese	NR	Concomitant intertrochanteric osteotomy in 16 hips	63/75	58	29.0 ± 12; 13.0–56.0	22.0 ± 3.0; 16.0–28.0	NR
Levack AE [52]	2020	USA	*Bone & Joint Journal*	RCT	1	2014–2018	0.12	Bernese	Modified Smith–Petersen	HAS: 23	81/81	78	25.9 ± 7.4; 14.0–46.0	23.5 ± 4.0; 16.3–36.8	NR
								Bernese	Modified Smith–Petersen	HAS: 13	40/40	38	25.1 ± 7.2; 15.0–42.0	23.9 ± 4.5; 16.9–36.8	TXA
								Bernese	Modified Smith–Petersen	HAS: 10	41/41	40	26.8 ± 7.7; 14.0–46.0	23.1 ± 3.5; 16.3–31.8	Non‐TXA
Li C [53]	2022	China	*BMC Surgery*	Retrospective case series	4	2016–2019	2.93 ± 0.88; 1.5–4.7	Bernese	Modified Smith–Petersen/Bikini + posterolateral assist incision	NR	58/65	52	28.1 ± 8.4; 11.0–49.0	22.0 ± 3.0; 17.4–30.4	NR
Luo D [54]	2016	China	*Therapeutics and Clinical Risk Management*	Retrospective comparative study	3	2010–2011	NR	Bernese	Improved ilioinguinal approach; modified Smith–Petersen approach; two‐incision Smith–Petersen approach	Arthroscopy, femoral osteotomy, labrum repair	95/101	81	11.0–45.0	NR	NR
Luo R [55]	2021	China	*Journal of Orthopaedic Surgery and Research*	Retrospective comparative study	1	2014–2019	1.0	Bernese	Modified Smith–Petersen/ilioinguinal	NR	61/66	39	26.8 ± 8.3; 16.0–45.0	20.4 ± 1.2	NR
Ma S [56]	2022	China	*The International Journal of Artificial Organs*	Randomized comparative study	2	2016–2020	0.5	Bernese	Modified Smith–Petersen	NR	22/22	18	28.0; 16.0–36.0	NR	NR
Markhardt BK [57]	2021	USA	*Journal of Hip Preservation Surgery*	Retrospective comparative study	3	2017–2020	NR	Bernese	Anterior (bikini, rectus sparing)	NR	26/28	24	29.4 ± 8.6	25.8 ± 4.2	NR
Marshall A [58]	2025	Canada	*The Journal of Arthroplasty*	Retrospective comparative study	3	2015–2021	0.25	Bernese	Smith–Petersen/bikini	Hip arthroscopy: 27; Osteochondroplasty: 7; Subspine decompression: 18;	94/94	70	28.0; 16.0–46.0	25.8; 18.9–37.1	NR
McLawhorn AS [61]	2016	USA	*The Journal of Arthroplasty*	Retrospective cohort study	3	2011–2014	NR	Bernese	Modified Smith–Petersen	HAS: 22	93/93	89	24.2 ± 8.0	22.9 ± 3.4	NR
Peters CL [72]	2006	USA	*Journal of Bone and Joint Surgery*	Retrospective case series	4	1997–2003	3.89; 2.5–7.3	Bernese	Modified Smith–Petersen	Femoral osteotomy *n* = 13; arthrotomy *n* = 49; osteochondroplasty *n* = 35; labral resection *n* = 11	73/83	55	28.0; 15.0–47.0	28.5; 17.1–33.9	NR
Peters CL [73]	2015	USA	*Clinical Orthopaedics and Related Research*	Retrospective comparative study	3	2009–2013	NR	Bernese	Rectus takedown + rectus sparing (combined)	NR	75/75	55	24.0	23.5	NR
Pogliacomi F [74]	2005	Italy/Sweden	*Acta Orthopaedica*	Retrospective case series	4	1994–2001	4.1; 1.5–8.0	Bernese	Modified Smith–Petersen/ilioinguinal	US femoral osteotomy: 1; capsulotomy: 2	32/36	31	35.0; 15.0–55.0	NR	NR
Pulido LF [75]	2008	USA	*Journal of Surgical Orthopaedic Advances*	Retrospective case series	4	1996–2003	NR	Bernese	Modified Smith–Petersen	NR	107/108	84	30.0; 12.0–49.0	23.6	NR
Sabbag CM [82]	2019	USA	*The American Journal of Sports Medicine*	Prospective case series	4	2007–2016	3.0; 1.0–8.0	Bernese	NR	HAS	240/248	207	26.6 ± 9.2; 12.0–53.0	NR	NR
Shang JJ [83]	2020	China	*Orthopaedic Surgery*	Retrospective comparative study	3	2010–2018	NR	Bernese	NR	NR	307/307	307	28.7 ± 8.4	21.9 ± 3.1	NR
								Bernese	NR	NR	74/74	74	28.5 ± 7.6	21.9 ± 3.4	Non‐TXA
								Bernese	NR	Proximal femoral osteotomy	233/233	233	28.8 ± 8.6	21.9 ± 2.9	TXA
Shon HC [84]	2023	South Korea	*Archives of Orthopaedic and Trauma Surgery*	Retrospective case series	4	1997–2005	11.5; 8.0–16.0	Bernese	Smith–Petersen + Kocher–Langenbeck (dual approach)		49/53	46	39.9; 13.0–62.0	NR	NR
Siebenrock KA [85]	1999	Switzerland	*Clinical Orthopaedics and Related Research*	Retrospective case series	4	1991–1995	11.3	Bernese	Anterior (Smith–Petersen/ilioinguinal‐type)	Additional acetabular procedures: 13; femoral (abduction) osteotomy: 7	63/75	NR	29.0; 13.0–56.0	NR	NR
Sierra RJ [86]	2017	USA	*Journal of Hip Surgery*	Retrospective cohort study	3	1996–2009	10.0; 24.0–236.0	Bernese	Anterior‐based	HAS: 1	268/299	123	31.0; 12.0–56.0	NR	NR
Stambough JB [87]	2014	USA	*Clinical Orthopaedics and Related Research*	Retrospective multicenter cohort study	3	2008–2012	2.5 ± 1.1; 2.0–5.1	Bernese	Anterior‐based	Osteochondroplasty: 29; arthroscopy: 15; labral repair: 6; intertrochanteric osteotomy: 2; labral resection: 3	78/78	70	19.9 ± 6.0; 9.0–35.4	24.0 ± 4.3; 11.7–43.0	NR
Steppacher SD [88]	2008	Switzerland	*Clinical Orthopaedics and Related Research*	Retrospective case series	4	1984–2003	20.4 ± 1.1; 19.0–23.0	Bernese	Modified Smith–Petersen	Intertrochanteric osteotomy: 16	63/75	58	29.3 ± 11.6; 13.0–56.0	22.1 ± 3.1; 15.8–28.2	NR
Tang Y [95]	2022	China	*International Orthopaedics*	Retrospective case series	4	2019–2021	2.0; 1.28–2.32	Modifizierte Bernese	Combined posterior + anterior mini‐incisions	NR	34/34	32	38.7; 25.0–54.0	21.4 ± 0.7	NR
Thawrani D [96]	2010	USA	*Journal of Bone and Joint Surgery. American Volume*	Retrospective case series	4	NR	2.0	Bernese	Modified Smith–Petersen (abductor‐sparing)	NR	76/83	62	15.6 ± 2.4; 11.0–21.0	23.9 ± 4.9	NR
Troelsen A (1) [98]	2008	Denmark	*Acta Orthopaedica*	Retrospective comparative study	3	1998–2007	5.0; 1.0–9.2	Bernese	Ilioinguinal; minimally invasive (transsartorial)	NR	211/263	174	33.0	NR	NR
Troelsen A (2) [99]	2008	Denmark	*Journal of Bone and Joint Surgery. American Volume*	Retrospective case series	4	2003–2005	4.3; 2.0–4.3	Bernese	Minimally invasive transsartorial	NR	91/94	76	37.2	NR	NR
van der Merwe M [100]	2019	New Zealand	*Journal of Hip Preservation Surgery*	Retrospective comparative study	3	2016–2018	NR	Bernese	Modified Smith–Petersen	No	58/58	44	24.7; 17.6–30.2	24.6; 22.1–28.3	NR
								Bernese	Modified Smith–Petersen	No	40/40	33	24.7; 17.6–29.4	25.8; 23.4–28.3	Cell saver
								Bernese	Modified Smith–Petersen	No	18/18	11	23.8; 17.9–30.2	23.5; 22.1–27.7	No cell saver
Wassilew GI [102]	2015	Germany	*The Bone & Joint Journal*	Retrospective comparative study	3	NR	NR	Bernese	NR	No	96/96	84	29.6 ± 8.7	23.9 ± 4.4	NR
								Bernese	NR	No	48/48	42	27.4 ± 7.0	24.2 ± 4.7	TXA
								Bernese	NR	No	48/48	42	31.7 ± 10.1	23.5 ± 4.0	Non‐TXA
Wingerter SA [106]	2015	USA	*Clinical Orthopaedics and Related Research*	Retrospective comparative study	3	2011–2013	NR	Bernese	NR	HAS: 34	100/100	85	27.5; 14.0–49.0	23.5; 17.0–33.0	NR
								Bernese	NR	HAS: 23	50/50	44	27.0; 17.0–47.0	23	TXA
								Bernese	NR	HAS: 11	50/50	41	28.0; 13.0–49.0	24	Non‐TXA
Yilmaz M [111]	2022	Turkey	*European Review for Medical and Pharmacological Sciences*	Retrospective comparative study	3	2001–2015	7.53 ± 3.14; 6.0–15.0	Bernese	Modified Smith–Petersen	NR	43/43	31	32.2 ± 8.4; 19.0–45.0	26.0 ± 2.6; 18.9–29.4	NR
Zaltz I [112]	2014	USA	*Journal of Bone and Joint Surgery*	Prospective case series	4	2007–2009	1.2; 0.7–2.9	Ganz	Smith–Petersen	Femoral head–neck osteochondroplasty: 118; hip arthroscopy: 42;	205/205	143	25.4; 11.0–54.0	25.3; 11.7–46.6	NR
Zhu J [113]	2013	China	*International Orthopaedics*	Retrospective case series	4	2001–2009	5.1; 2.0–10.0	Bernese	Modified Smith–Petersen	Osteochondroplasty: 11; varus osteotomy: 4 hips; labrum debridement: 3 hips	36/41	34	39.5; 35.0–54.0	21.4; 18.2–26.6	NR
Ziran N [114]	2018	USA	*Journal of the American Academy of Orthopaedic Surgeons*	Retrospective case series	4	1987–2014	11.2; 2.0–27.0	Bernese	Modified Smith–Petersen	NR	258/302	215	32.8; 13.0–63.0	NR	NR

*Note*: Summary of included studies reporting study design, sample size, patient demographics (age, sex and BMI), surgical technique, concomitant procedures and follow‐up duration.

Abbreviations: BMI, body mass index; FAI, femoroacetabular impingement; HAS, hip arthroscopy; NR, not reported; SD, standard deviation; TXA, tranexamic acid.

Across the included studies, a total of 4402 patients and 4767 hips were analyzed, with a clear predominance of female patients. Mean patient age generally ranged from adolescence to early adulthood, and BMI values were typically within the non‐obese range, reflecting the standard PAO population (Table 1).

The Bernese PAO was the predominant surgical technique across studies. Most procedures were performed using anterior‐based approaches, particularly the modified Smith‐Petersen approach, although variations such as minimally invasive, bikini and abductor‐sparing techniques were also reported. Concomitant procedures were common and included femoral osteochondroplasty, hip arthroscopy, labral treatment and femoral osteotomy, highlighting the procedural complexity and heterogeneity of surgical management in this population (Table 1).

### Quality and publication bias assessment

Risk of bias was assessed in 45 non‐randomized studies [2, 3, 7, 8, 11, 13, 14, 16, 24, 32, 33, 39, 40, 44, 46, 47, 51, 53, 54, 55, 57, 58, 61, 72, 73, 74, 75, 82, 83, 84, 85, 86, 87, 88, 95, 96, 98, 99, 100, 102, 106, 111, 112, 113, 114] using the ROBINS‐I tool and in two randomized controlled trials [52, 56] using the RoB 2 tool. Among the non‐randomized studies, overall risk of bias was rated as moderate in 2 studies [3, 111], serious in 27 studies [2, 7, 11, 13, 32, 33, 40, 46, 54, 55, 57, 58, 61, 73, 75, 83, 86, 87, 95, 96, 98, 100, 102, 106, 112, 113, 114], and critical in 16 studies [8, 14, 16, 24, 39, 44, 47, 51, 53, 72, 74, 82, 84, 85, 88, 99] (Table 2). Confounding was the most frequently affected domain and was commonly rated as serious or critical, whereas intervention classification and deviations from intended interventions were predominantly rated as low risk. The domains of participant selection, missing data, outcome measurement, and selective reporting were generally rated as low to moderate, although several studies showed more substantial limitations in these domains (Table 2). The two randomized controlled trials [52, 53, 54, 55, 56] demonstrated an overall low risk of bias, with low risk across all domains except for selective reporting, which was rated as some concerns (Table 2).

**Table 2:** Risk‐of‐bias assessment using ROBINS‐I and RoB 2.

Study/non‐RCTs	Confounding	Selection	Intervention classification	Deviations	Missing data	Outcome measurement	Reporting	Overall
Albers 2013 [2]	Serious	Moderate	Moderate	Low	Low	Low	Moderate	Serious
Amano 2014 [3]	Moderate	Moderate	Low	Low	Low	Low	Moderate	Moderate
Atwal 2008 [7]	Serious	Moderate	Low	Low	Low	Moderate	Moderate	Serious
Baraka 2022 [8]	Critical	Serious	Low	Low	Low	Moderate	Moderate	Critical
Bernstein 2007 [11]	Serious	Moderate	Low	Low	Moderate	Moderate	Moderate	Serious
Bryan 2016 [13]	Serious	Moderate	Low	Low	Low	Moderate	Moderate	Serious
Burke 2011 [14]	Critical	Serious	Low	Low	Low	Moderate	Moderate	Critical
Clohisy 2005 [16]	Critical	Serious	Low	Low	Low	Moderate	Moderate	Critical
Domb 2015 [24]	Critical	Serious	Low	Low	Moderate	Moderate	Moderate	Critical
Guo 2024 [32]	Serious	Moderate	Low	Low	Low	Moderate	Moderate	Serious
Haertlé 2024 [33]	Serious	Low	Low	Low	Low	Moderate	Low	Serious
Khan 2017 [39]	Critical	Moderate	Low	Low	Moderate	Moderate	Moderate	Critical
Kim 2009 [40]	Serious	Moderate	Low	Low	Low	Moderate	Moderate	Serious
Kraeutler 2018 [44]	Critical	Moderate	Low	Low	Low	Moderate	Moderate	Critical
Lee 2013 [46]	Serious	Low	Low	Low	Low	Moderate	Low	Serious
Lee SH 2025 [47]	Critical	Serious	Low	Low	Low	Moderate	Moderate	Critical
Lerch 2017 [51]	Critical	Serious	Low	Moderate	Moderate	Moderate	Moderate	Critical
Li 2022 [53]	Critical	Serious	Low	Low	Moderate	Moderate	Moderate	Critical
Luo D 2016 [54]	Serious	Moderate	Low	Low	Moderate	Moderate	Moderate	Serious
Luo R 2021 [55]	Serious	Moderate	Low	Low	Low	Moderate	Moderate	Serious
Markhardt BK 2021 [57]	Serious	Moderate	Low	Low	Low	Moderate	Moderate	Serious
Marshall A 2025 [58]	Serious	Moderate	Low	Low	Low	Moderate	Moderate	Serious
McLawhorn AS 2016 [61]	Serious	Moderate	Low	Low	Low	Moderate	Moderate	Serious
Peters CL 2006 [72]	Critical	Serious	Low	Moderate	Moderate	Moderate	Moderate	Critical
Peters CL 2015 [73]	Serious	Moderate	Low	Low	Low	Moderate	Moderate	Serious
Pogliacomi 2005 [74]	Critical	Serious	Low	Moderate	Moderate	Moderate	Moderate	Critical
Pulido LF 2008 [75]	Serious	Moderate	Low	Low	Low	Moderate	Moderate	Serious
Sabbag CM 2019 [82]	Critical	Moderate	Low	Low	Low	Moderate	Moderate	Critical
Shang JJ 2020 [83]	Serious	Moderate	Low	Moderate	Low	Moderate	Moderate	Serious
Shon HC 2023 [84]	Critical	Serious	Low	Low	Moderate	Moderate	Moderate	Critical
Siebenrock KA 1999 [85]	Critical	Serious	Low	Moderate	Moderate	Moderate	Moderate	Critical
Sierra RJ 2017 [86]	Serious	Moderate	Low	Low	Moderate	Moderate	Moderate	Serious
Stambough JB 2014 [87]	Serious	Moderate	Low	Low	Moderate	Low	Moderate	Serious
Steppacher SD 2008 [88]	Critical	Serious	Low	Low	Moderate	Moderate	Moderate	Critical
Tang Y 2022 [95]	Serious	Moderate	Low	Low	Low	Moderate	Moderate	Serious
Thawrani D 2010 [96]	Serious	Moderate	Low	Moderate	Low	Moderate	Moderate	Serious
Troelsen A et al. 2008 (1) [98]	Serious	Moderate	Low	Moderate	Moderate	Moderate	Moderate	Serious
Troelsen A et al. 2008 (2) [99]	Critical	Serious	Low	Low	Moderate	Moderate	Moderate	Critical
van der Merwe M et al. 2019 [100]	Serious	Moderate	Low	Low	Low	Moderate	Moderate	Serious
Wassilew GI et al. 2015 [102]	Serious	Moderate	Low	Low	Low	Moderate	Moderate	Serious
Wingerter SA et al. 2015 [106]	Serious	Moderate	Low	Low	Low	Moderate	Moderate	Serious
Yilmaz M et al. 2022 [111]	Moderate	Moderate	Low	Low	Low	Moderate	Moderate	Moderate
Zaltz I et al. 2014 [112]	Serious	Moderate	Low	Low	Moderate	Low	Low	Serious
Zhu J et al. 2013 [113]	Serious	Moderate	Low	Low	Moderate	Moderate	Moderate	Serious
Ziran N et al. 2019 [114]	Serious	Serious	Low	Low	Serious	Moderate	Moderate	Serious

Study/RCT	Randomization	Deviations	Missing data	Outcome measurement	Reporting	Overall
Levack AE et al. 2020 [52]	Low	Low	Low	Low	Some concerns	Low
Ma S 2022 [56]	Low	Low	Low	Low	Some concerns	Low

*Note*: Risk‐of‐bias assessment for included studies using the ROBINS‐I tool for non‐randomized studies and the RoB 2 tool for randomized trials. Studies are categorized as low, moderate, serious or critical risk of bias across domains, with confounding identified as the predominant source of bias.

Abbreviations: RCT, randomized controlled trial; RoB 2, Risk of Bias 2 tool; ROBINS‐I, Risk of Bias In Non‐randomized Studies of Interventions.

Visual inspection of funnel plots was performed for all pooled outcomes (Figures [Link], [Link], [Link], [Link], [Link], [Link]). However, interpretation of funnel plot asymmetry should be made cautiously because several analyses included only a limited number of studies, and substantial between‐study heterogeneity was present across most outcomes. Therefore, no definitive conclusions regarding publication bias or small‐study effects can be drawn.

### Pooled perioperative blood management outcomes

**Figure 2:**
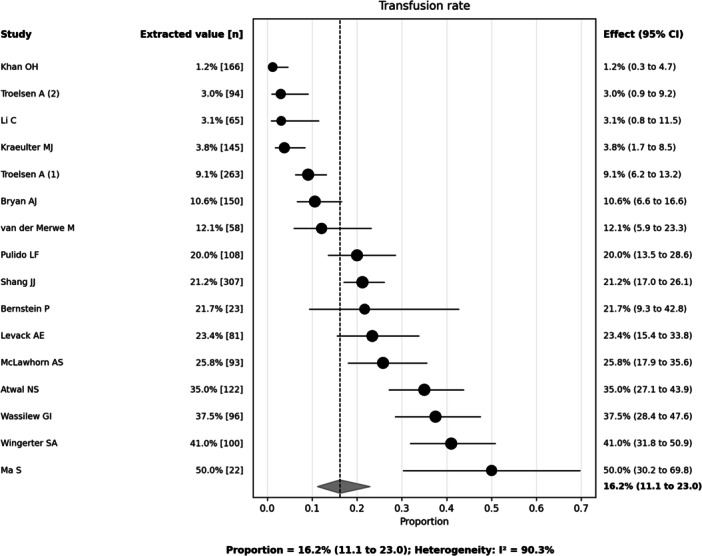
Forest plot of the pooled transfusion rate following periacetabular osteotomy (PAO). A random‐effects meta‐analysis of proportions was performed to estimate the overall transfusion rate across included studies. Effect estimates are presented as proportions with 95% confidence intervals (CIs).

**Figure 3:**
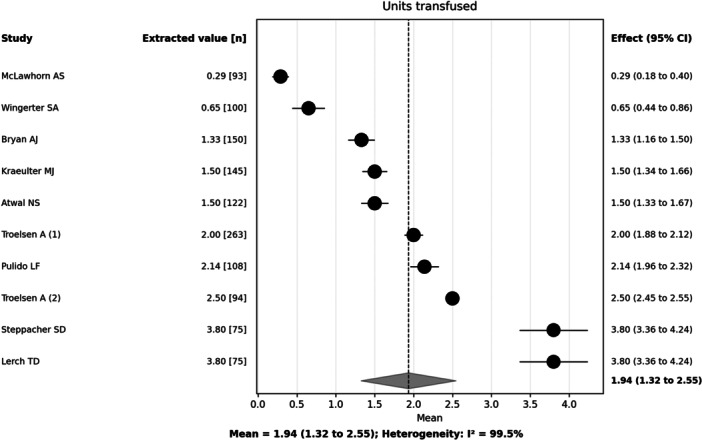
Forest plot of the pooled mean number of transfused units following periacetabular osteotomy (PAO). A random‐effects meta‐analysis of means was performed across included studies. Effect estimates are presented as mean values with 95% confidence intervals (CIs).

**Figure 4:**
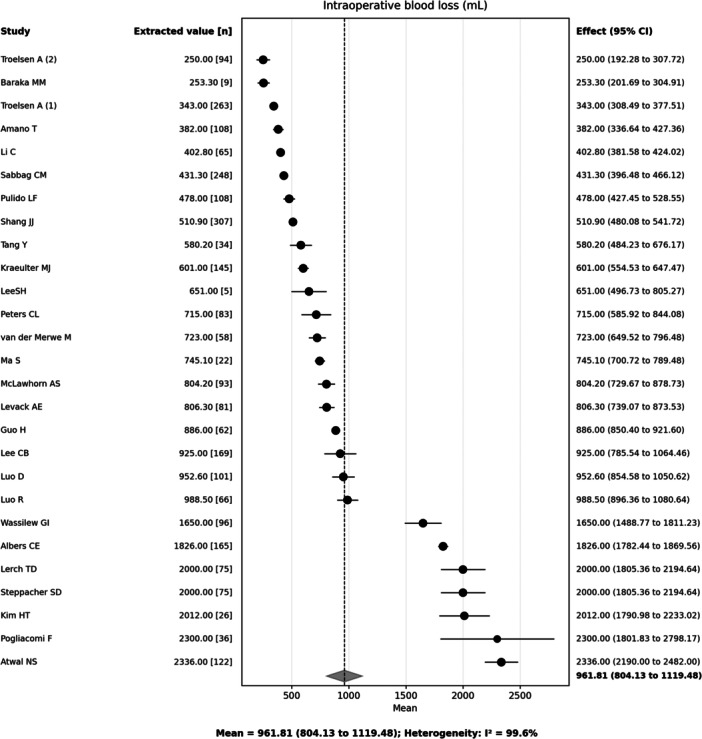
Forest plot of the pooled mean intraoperative blood loss following periacetabular osteotomy (PAO). A random‐effects meta‐analysis of means was performed across included studies. Effect estimates are presented in millilitres (mL) with 95% confidence intervals (CIs).

**Figure 5:**
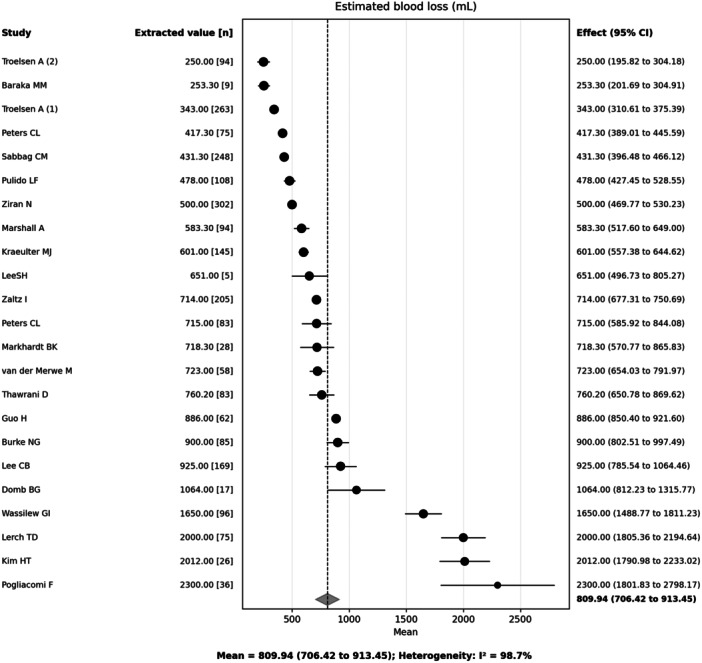
Forest plot of the pooled mean estimated blood loss following periacetabular osteotomy (PAO). A random‐effects meta‐analysis of means was performed across included studies. Effect estimates are presented in millilitres (mL) with 95% confidence intervals (CIs).

**Figure 6:**
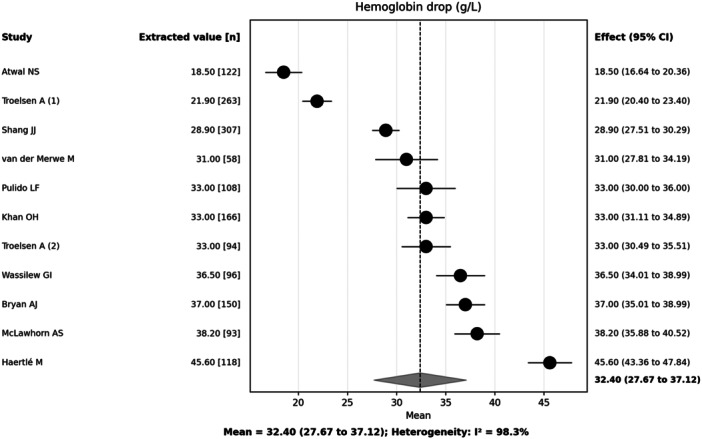
Forest plot of the pooled mean perioperative haemoglobin drop following periacetabular osteotomy (PAO). A random‐effects meta‐analysis of means was performed across included studies. Effect estimates are presented in g/L with 95% confidence intervals (CIs).

**Figure 7:**
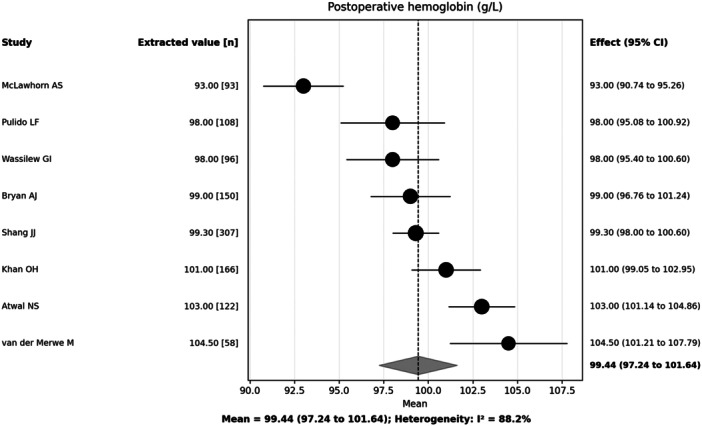
Forest plot of the pooled mean postoperative haemoglobin level following periacetabular osteotomy (PAO). A random‐effects meta‐analysis of means was performed across included studies. Effect estimates are presented in g/L with 95% confidence intervals (CIs).

Across the included studies, the pooled transfusion rate following PAO was 16.2% (95% CI = 11.1–23.0), with substantial between‐study heterogeneity (*I* ^2^ = 90.3%) (Figure 2). The pooled mean number of transfused units per patient was 1.94 units (95% CI = 1.32–2.55; *I* ^2^ = 99.5%) (Figure 3). The pooled mean intraoperative blood loss was 961.8 mL (95% CI = 804.1–1119.5; *I* ^2^ = 99.6%) (Figure 4), whereas the pooled mean estimated blood loss was 809.9 mL (95% CI = 706.4–913.5; *I* ^2^ = 98.7%) (Figure 5). The pooled mean haemoglobin drop was 32.4 g/L (95% CI = 27.7–37.1; *I* ^2^ = 98.3%) (Figure 6), and the pooled mean postoperative haemoglobin level was 99.4 g/L (95% CI = 97.2–101.6; *I* ^2^ = 88.2%) (Figure 7).

### Meta‐regression of influencing factors

Univariable meta‐regression analyses were performed to explore study‐level factors potentially associated with perioperative blood management outcomes after PAO (Table 3). Investigated moderators included female sex, mean age, BMI, operative time, length of stay, complication rate, reoperation rate, THA conversion rate, TXA use, cell saver use, autologous predonation and preoperative haemoglobin (Table 3).

**Table 3:** Univariable meta‐regression of study‐level moderators for perioperative blood management outcomes following periacetabular osteotomy (PAO).

Outcome	Moderator	*k*	*β* (95% CI)	p‐value
Transfusion rate proportion	TXA use	11	−1.34 (−3.33 to 0.66)	0.163
	Autologous predonation	7	0.44 (−0.93 to 1.82)	0.444
	Preop haemoglobin	9	−0.00 (−0.02 to 0.02)	0.929
	Operation time	12	0.00 (−0.01 to 0.01)	0.473
	Mean age	15	−0.15 (−0.29 to −0.01)	**0.035**
	BMI	9	0.14 (−0.51 to 0.79)	0.631
	Female sex proportion	15	2.64 (−1.55 to 6.83)	0.197
	Length of stay	7	−0.03 (−0.46 to 0.41)	0.880
	Complications proportion	10	−6.46 (−26.52 to 13.60)	0.479
	Reoperations proportion	4	−29.25 (−118.60 to 60.11)	0.294
	THA conversion proportion	5	0.44 (−0.04 to 0.92)	0.062
Units transfused mean	TXA use	8	−0.58 (−3.86 to 2.70)	0.681
	Preop haemoglobin	5	0.12 (−0.14 to 0.38)	0.234
	Operation time	7	−0.01 (−0.02 to −0.00)	**0.016**
	Mean age	10	0.13 (0.07 to 0.20)	**0.001**
	BMI	7	0.11 (−0.82 to 1.05)	0.770
	Female sex proportion	10	−6.52 (−13.74 to 0.71)	0.071
	Length of stay	4	0.55 (0.18 to 0.92)	**0.024**
	Complications proportion	5	−46.75 (−219.64 to 126.13)	0.453
	THA conversion proportion	5	−0.31 (−0.89 to 0.27)	0.188
Intraoperative blood loss (mL)	TXA use	21	−237.42 (−642.56 to 167.72)	0.235
	Autologous predonation	7	−196.17 (−722.62 to 330.28)	0.382
	Preop haemoglobin	8	−2.57 (−12.62 to 7.48)	0.555
	Operation time	24	4.36 (1.30 to 7.42)	**0.007**
	Mean age	26	−23.42 (−72.73 to 25.90)	0.337
	BMI	18	62.74 (−131.40 to 256.88)	0.503
	Female sex proportion	27	−1041.79 (−2477.90 to 394.32)	0.148
	Length of stay	9	−171.20 (−416.72 to 74.32)	0.143
	Complications proportion	16	142.57 (−1219.94 to 1505.08)	0.826
	Reoperations proportion	7	−1474.01 (−6524.21 to 3576.19)	0.487
	THA conversion proportion	10	6474.86 (2491.75 to 10457.98)	**0.006**
Estimated blood loss (mL)	TXA use	18	132.95 (−157.15 to 423.06)	0.346
	Autologous predonation	4	−103.58 (−1943.10 to 1735.94)	0.831
	Preop haemoglobin	4	−14.75 (−285.74 to 256.24)	0.837
	Operation time	21	2.22 (0.19 to 4.24)	**0.034**
	Mean age	23	−13.34 (−38.28 to 11.59)	0.278
	BMI	15	−35.09 (−123.06 to 52.88)	0.404
	Female sex proportion	23	675.89 (−1239.19 to 2590.97)	0.471
	Length of stay	9	−7.34 (−86.97 to 72.28)	0.834
	Complications proportion	16	632.23 (−368.31 to 1632.77)	0.197
	Reoperations proportion	10	−1263.00 (−3756.37 to 1230.37)	0.276
	THA conversion proportion	10	3043.60 (673.15 to 5414.05)	**0.018**
Haemoglobin drop (g/L)	TXA use	8	13.33 (−5.64 to 32.30)	0.136
	Autologous predonation	5	1.40 (−22.69 to 25.49)	0.865
	Preop haemoglobin	9	0.99 (0.43 to 1.56)	**0.004**
	Operation time	10	0.05 (−0.07 to 0.17)	0.384
	Mean age	11	−0.43 (−1.81 to 0.96)	0.502
	BMI	7	2.46 (−0.67 to 5.59)	0.099
	Female sex proportion	11	4.84 (−44.91 to 54.59)	0.831
	Length of stay	7	1.06 (−5.59 to 7.70)	0.699
	Complications proportion	6	29.91 (−16.13 to 75.95)	0.146
	Reoperations proportion	4	−146.76 (−1397.29 to 1103.78)	0.664
	THA conversion proportion	4	−435.77 (−1672.78 to 801.24)	0.269
Postop haemoglobin (g/L)	TXA use	5	5.06 (−17.62 to 27.73)	0.529
	Autologous predonation	5	−6.70 (−22.00 to 8.60)	0.258
	Preop haemoglobin	8	−0.16 (−0.74 to 0.42)	0.521
	Operation time	7	−0.04 (−0.09 to 0.02)	0.136
	Mean age	8	−0.01 (−1.02 to 1.00)	0.980
	BMI	6	0.24 (−2.30 to 2.78)	0.806
	Female sex proportion	8	−9.58 (−39.60 to 20.44)	0.465
	Length of stay	4	0.18 (−6.97 to 7.33)	0.923

*Note*: Regression coefficients (β) with 95% confidence intervals (CIs) and *p* values are shown for the association between prespecified study‐level moderators and pooled perioperative blood management outcomes. Continuous moderators were analyzed using reported study‐level means, whereas count‐based variables were converted to proportions where appropriate. Binary moderators were coded dichotomously. Results should be interpreted as exploratory due to the observational nature of the included data and the limited number of studies available for several analyses. Statistically significant results are highlighted in bold.

Abbreviations: BMI, body mass index; THA, total hip arthroplasty; TXA, tranexamic acid.

Among the evaluated moderators, longer operative time was significantly associated with higher intraoperative blood loss (*β* = 4.36 mL/min, 95% CI = 1.30–7.42; *p* = 0.007) and higher estimated blood loss (β = 2.22 mL/min, 95% CI = 0.19–4.24; *p* = 0.034) (Table 3). Higher preoperative haemoglobin was significantly associated with a greater perioperative haemoglobin drop (*β* = 0.99 g/L per 1 g/L increase, 95% CI = 0.43–1.56; *p* = 0.004) (Table 3). Higher mean age was significantly associated with a greater number of transfused units (*β* = 0.13, 95% CI = 0.07–0.20; *p* = 0.001), while older age was inversely associated with transfusion rate (*β* = −0.15, 95% CI = −0.29 to −0.01; *p* = 0.035) (Table 3). In addition, higher THA conversion rates were significantly associated with both increased intraoperative blood loss (*β* = 6474.86, 95% CI = 2491.75–10,457.98; *p* = 0.006) and increased estimated blood loss (*β* = 3043.60, 95% CI = 673.15–5414.05; *p* = 0.018) (Table 3).

## DISCUSSION

### Principal findings

The main finding of this study is that PAO is associated with substantial perioperative blood loss and a relevant transfusion burden. Across the included studies, pooled estimates were 16.2% for transfusion rate, 1.94 transfused units per patient, 961.8 mL for intraoperative blood loss and 809.9 mL for estimated blood loss. Univariable meta‐regression suggested that longer operative time was associated with higher intraoperative and estimated blood loss, higher preoperative haemoglobin was associated with greater haemoglobin drop and higher age was associated with transfusion‐related outcomes.

### Comparison with previous PAO blood management literature

The present findings expand the current PAO blood management literature. Previous meta‐analyses have primarily focused on the effectiveness of antifibrinolytic strategies rather than on the overall perioperative blood loss profile of PAO itself. Wang et al. [101] reported that antifibrinolytic agents were associated with reduced total blood loss, decline in postoperative haemoglobin levels, transfusion rates, and length of stay after PAO. In contrast to these intervention‐focused analyses, which primarily evaluated the effectiveness of antifibrinolytic strategies such as TXA, the present study was designed to establish pooled benchmark estimates for perioperative blood loss and transfusion burden across the broader PAO literature [101, 110], independent of any single intervention. This benchmark‐oriented approach represents a distinct contribution compared to prior PAO meta‐analyses focused on specific interventions. While previous studies have demonstrated beneficial effects of TXA in PAO [101], no consistent association between TXA use and the predefined blood management outcomes was observed in the present meta‐regression.

### Comparison with hip arthroplasty literature

The moderator pattern identified in the present study is also broadly consistent with the hip arthroplasty literature. In a prospective observational study of total hip and knee arthroplasty, Carling et al. [15] reported an overall transfusion rate of 16%, with estimated blood loss of 984 mL in hip arthroplasty patients, and identified preoperative haemoglobin as the main independent predictor of transfusion in hip patients. These findings are broadly comparable to the benchmark estimates observed in the present PAO synthesis, particularly with regard to transfusion burden, while the absolute magnitude of blood loss remains within a clinically similar range. Although PAO and THA differ substantially in patient age, indication, and surgical philosophy, the overall magnitude of perioperative blood loss appears clinically comparable.

Similarly, the meta‐analysis by Peng and Qiao [71] identified advanced age, female sex, preoperative anaemia, low BMI and increased intraoperative bleeding as risk factors for perioperative transfusion in THA. While not all of these variables emerged as significant moderators in the present PAO‐based meta‐regression, the observed association between higher age and a greater number of transfused units, as well as the clear association between longer operative time and higher blood loss, are directionally consistent with this broader arthroplasty literature [15, 71]. Taken together, these parallels suggest that at least part of the blood loss variability seen after PAO reflects general perioperative blood management principles rather than being entirely procedure‐specific.

### Interpretation of moderator findings

Among the investigated moderators, operative time emerged as the most consistent correlate of perioperative blood loss. Longer procedures were associated with both higher intraoperative blood loss and higher estimated blood loss, which is biologically plausible and clinically intuitive. Prolonged surgical exposure, more extensive soft‐tissue handling, and greater technical complexity likely contribute directly to cumulative bleeding. This supports the assumption that procedure‐related factors remain central drivers of perioperative blood loss in PAO.

Higher preoperative haemoglobin was associated with greater haemoglobin drop. This should not be interpreted as a harmful effect of higher baseline haemoglobin, but rather as a reflection of the mathematical and physiological fact that patients starting from a higher preoperative value can demonstrate a larger absolute decline. Likewise, the association between older age and a greater number of transfused units is clinically plausible and aligns with broader orthopaedic experience, where reduced physiological reserve and more restrictive tolerance of perioperative anaemia may contribute to transfusion decisions [15, 71].

In contrast, no consistent associations were observed for BMI, female sex, TXA use, cell saver use or autologous predonation in the present meta‐regression models. These findings should be interpreted with caution, as the analyses were based on aggregate study‐level data and are exploratory in nature.

### Relation to broader PAO literature

Beyond blood management specifically, the present findings fit well within the broader PAO literature, which is characterized by substantial heterogeneity in indication, surgical strategy and perioperative care. This has also been evident in other recent quantitative syntheses, such as the multilevel meta‐analysis comparing PAO and hip arthroscopy in borderline dysplasia [77]. That work demonstrated how heterogeneity in study design, patient selection and treatment definitions complicates quantitative interpretation in hip preservation research [77]. The same issue applies here: blood management outcomes after PAO are likely influenced not only by specific interventions such as TXA, but also by differences in surgical complexity, patient morphology, surgeon experience and institutional transfusion practice.

### Clinical implications

The present pooled estimates are clinically relevant because they may provide clinically useful benchmark estimates for perioperative blood loss and transfusion burden after PAO. These data may support preoperative counselling, perioperative planning and institutional blood management protocols. In addition, the identification of operative time as a key correlate of blood loss suggests that surgical complexity and efficiency remain important practical determinants of perioperative burden. The findings should be interpreted alongside existing evidence on blood conservation strategies such as TXA, which have demonstrated benefits in PAO‐specific comparative analyses [101].

### Limitations

Several limitations must be acknowledged. First, the available evidence was derived predominantly from retrospective, non‐randomized studies with moderate to critical risk of bias. Second, statistical heterogeneity was substantial across most pooled outcomes, which limits the precision and generalizability of the benchmark estimates. Third, moderator analyses were based on aggregate study‐level data and should therefore be regarded as exploratory and hypothesis‐generating and should not be interpreted as causal. Fourth, reporting of several potential moderators was inconsistent, and some variables could not be assessed robustly across all outcomes. Finally, variability in transfusion thresholds, perioperative protocols and definitions of blood loss parameters across studies may have contributed to between‐study heterogeneity and should be considered when interpreting the pooled estimates.

## CONCLUSION

PAO is associated with substantial perioperative blood loss and a relevant transfusion burden. These pooled estimates may provide clinically useful benchmark estimates for perioperative blood management.

## AUTHOR CONTRIBUTIONS

Nikolai Ramadanov and Jonathan Lettner performed the literature search, the data extraction and the risk of bias assessment. Nikolai Ramadanov conducted the statistical calculations. Nikolai Ramadanov created all figures and tables. Nikolai Ramadanov wrote the manuscript. Roland Becker, Robert Prill, Marko Ostojic and Sufian S. Ahmad supervised the work.

## CONFLICT OF INTEREST STATEMENT

The authors declare no conflicts of interest.

## ETHICS STATEMENT

The authors have nothing to report.

## Supporting information

Figure S1. Transfusion rate. Funnel plot of the studies included in the meta‐analysis of transfusion rate following periacetabular osteotomy (PAO). Visual inspection was performed to explore potential small‐study effects and publication bias.

Figure S2. Units transfused. Funnel plot of the studies included in the meta‐analysis of units transfused following periacetabular osteotomy (PAO). Visual inspection was performed to explore potential small‐study effects and publication bias.

Figure S3. Intraoperative blood loss. Funnel plot of the studies included in the meta‐analysis of intraoperative blood loss following periacetabular osteotomy (PAO). Visual inspection was performed to explore potential small‐study effects and publication bias.

Figure S4. Estimated blood loss. Funnel plot of the studies included in the meta‐analysis of estimated blood loss following periacetabular osteotomy (PAO). Visual inspection was performed to explore potential small‐study effects and publication bias.

Figure S5. Hemoglobin drop. Funnel plot of the studies included in the meta‐analysis of hemoglobin drop following periacetabular osteotomy (PAO). Visual inspection was performed to explore potential small‐study effects and publication bias.

Figure S6. Postoperative hemoglobin levels. Funnel plot of the studies included in the meta‐analysis of postoperative hemoglobin levels following periacetabular osteotomy (PAO). Visual inspection was performed to explore potential small‐study effects and publication bias.

Table S1. PRISMA Checklist.

## Data Availability

Available from the corresponding author upon reasonable request.
