# Supplementary figures and images for: Substantial blood loss and transfusion burden after periacetabular osteotomy: Benchmark estimates from a systematic review and meta‐analysis
Source: Knee Surg Sports Traumatol Arthrosc. 2026 Jun 3;34(7):2576–601. doi: 10.1002/ksa.70432 (PMC13327451; doi:10.1002/ksa.70432)

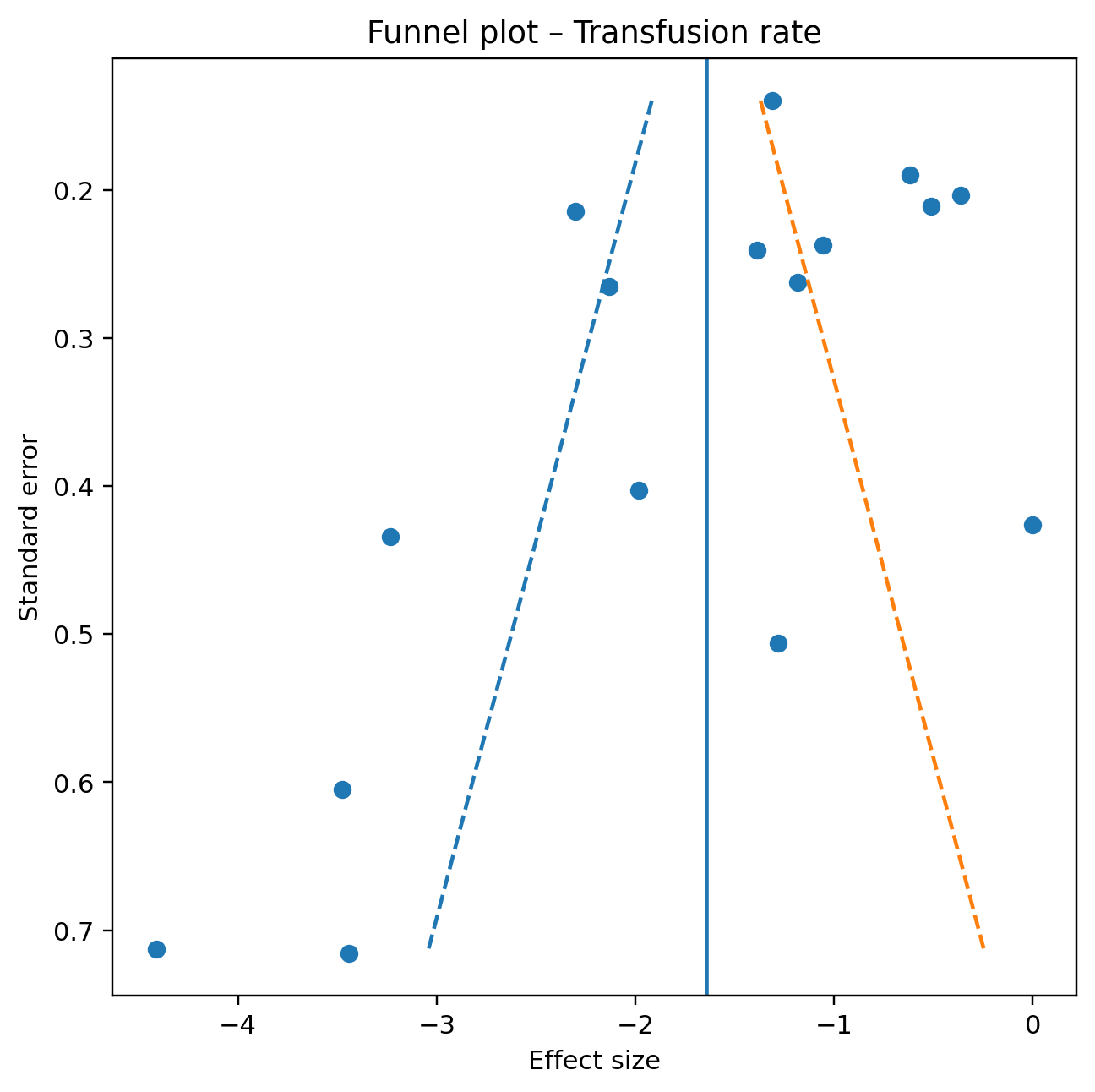

Supplement: Supplementary file 1 — Figure S1. Transfusion rate. Funnel plot of the studies included in the meta‐analysis of transfusion rate following periacetabular osteotomy (PAO). Visual inspection was performed to explore potential small‐study effects and publication bias. [file KSA-34-2576-s005.png]

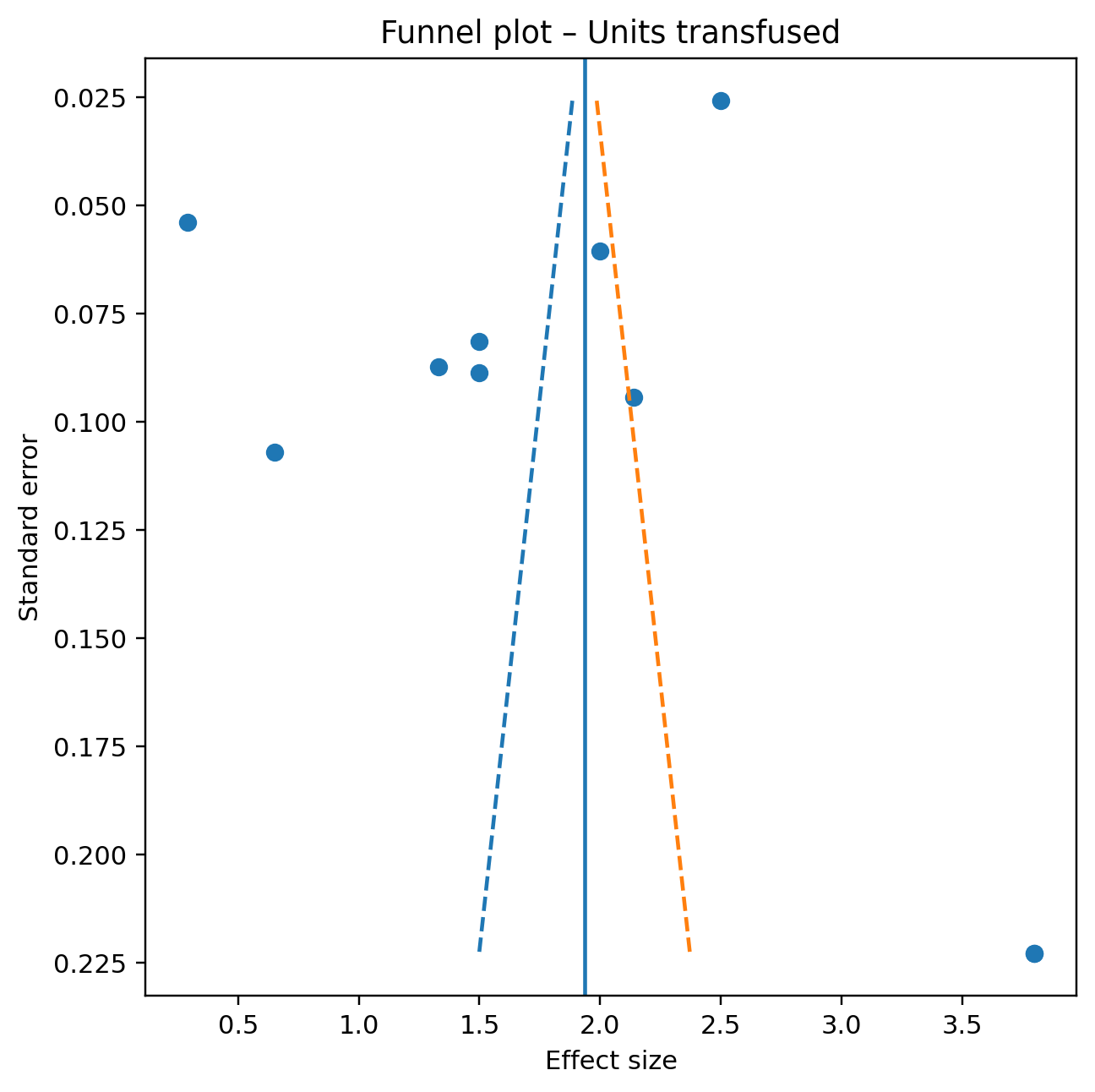

Supplement: Supplementary file 2 — Figure S2. Units transfused. Funnel plot of the studies included in the meta‐analysis of units transfused following periacetabular osteotomy (PAO). Visual inspection was performed to explore potential small‐study effects and publication bias. [file KSA-34-2576-s001.png]

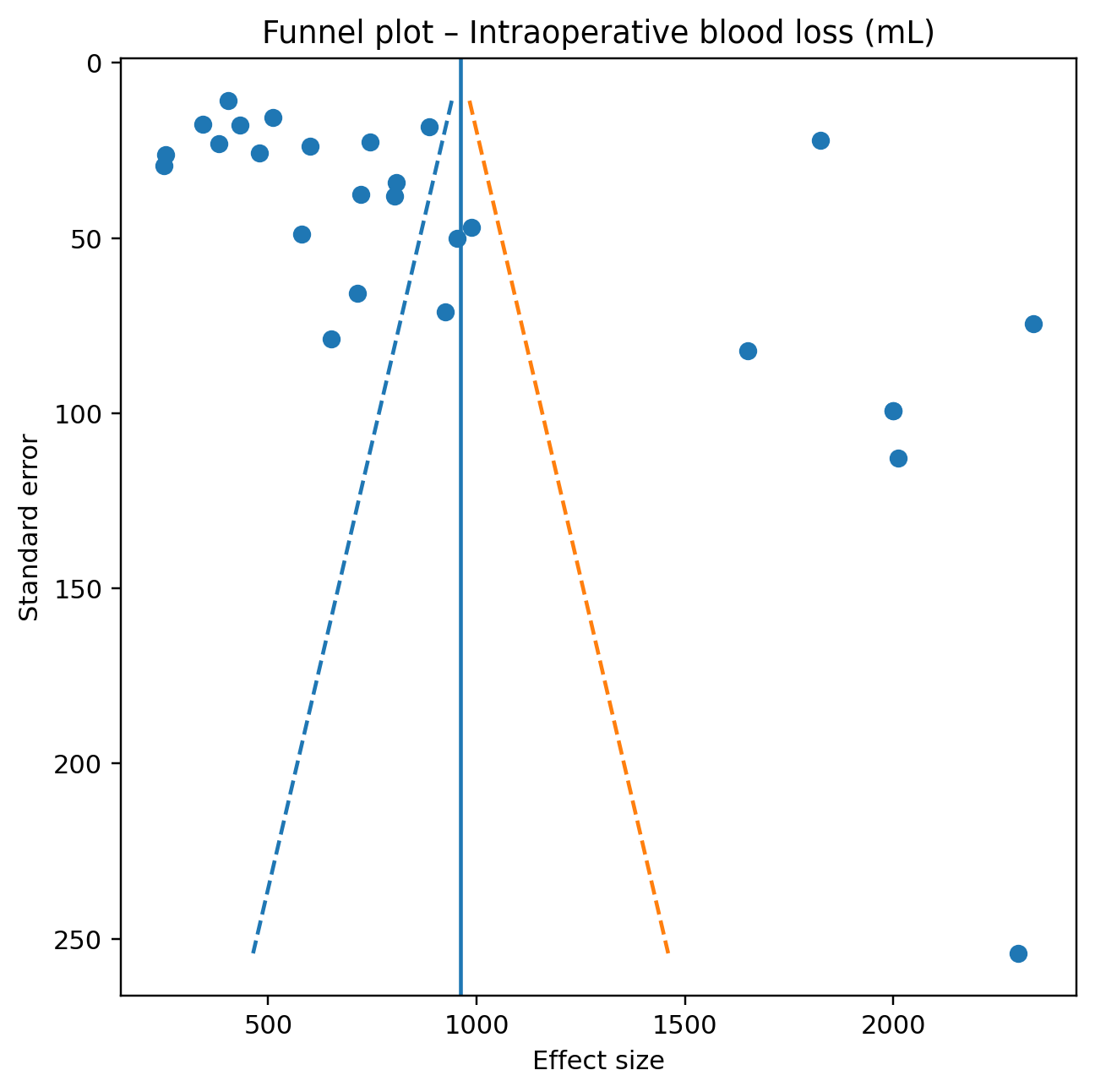

Supplement: Supplementary file 3 — Figure S3. Intraoperative blood loss. Funnel plot of the studies included in the meta‐analysis of intraoperative blood loss following periacetabular osteotomy (PAO). Visual inspection was performed to explore potential small‐study effects and publication bias. [file KSA-34-2576-s002.png]

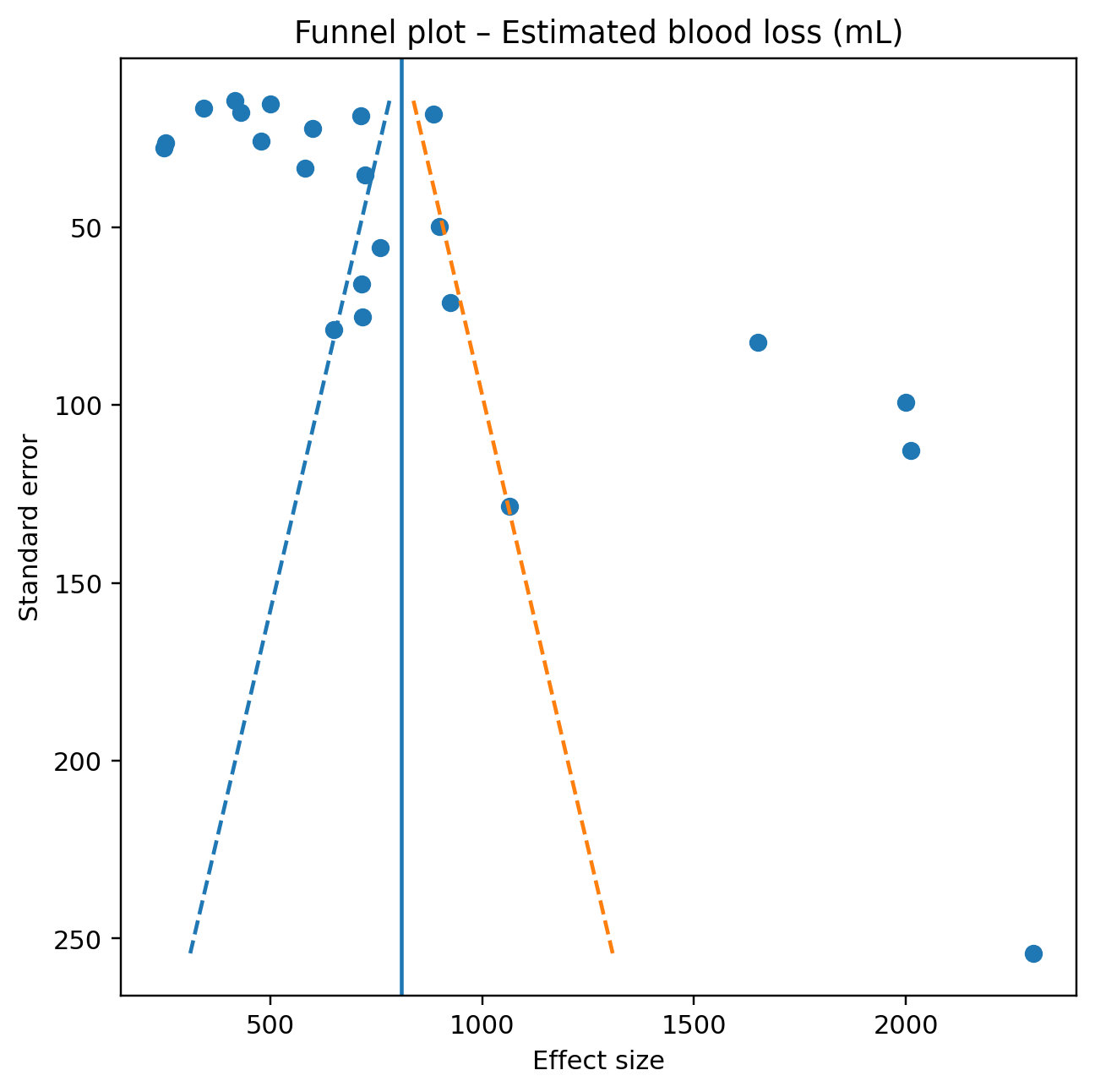

Supplement: Supplementary file 4 — Figure S4. Estimated blood loss. Funnel plot of the studies included in the meta‐analysis of estimated blood loss following periacetabular osteotomy (PAO). Visual inspection was performed to explore potential small‐study effects and publication bias. [file KSA-34-2576-s004.png]

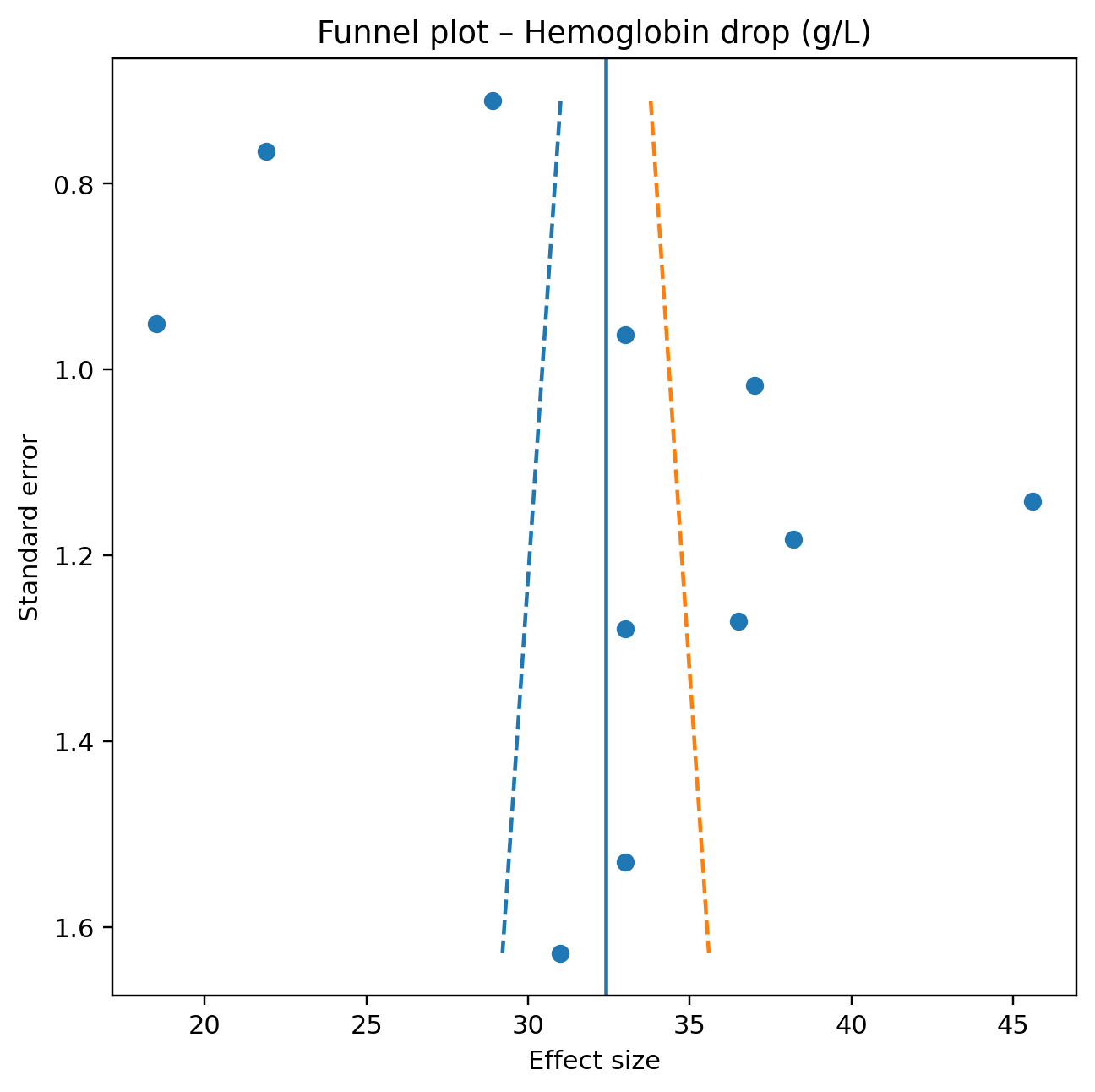

Supplement: Supplementary file 5 — Figure S5. Hemoglobin drop. Funnel plot of the studies included in the meta‐analysis of hemoglobin drop following periacetabular osteotomy (PAO). Visual inspection was performed to explore potential small‐study effects and publication bias. [file KSA-34-2576-s007.png]

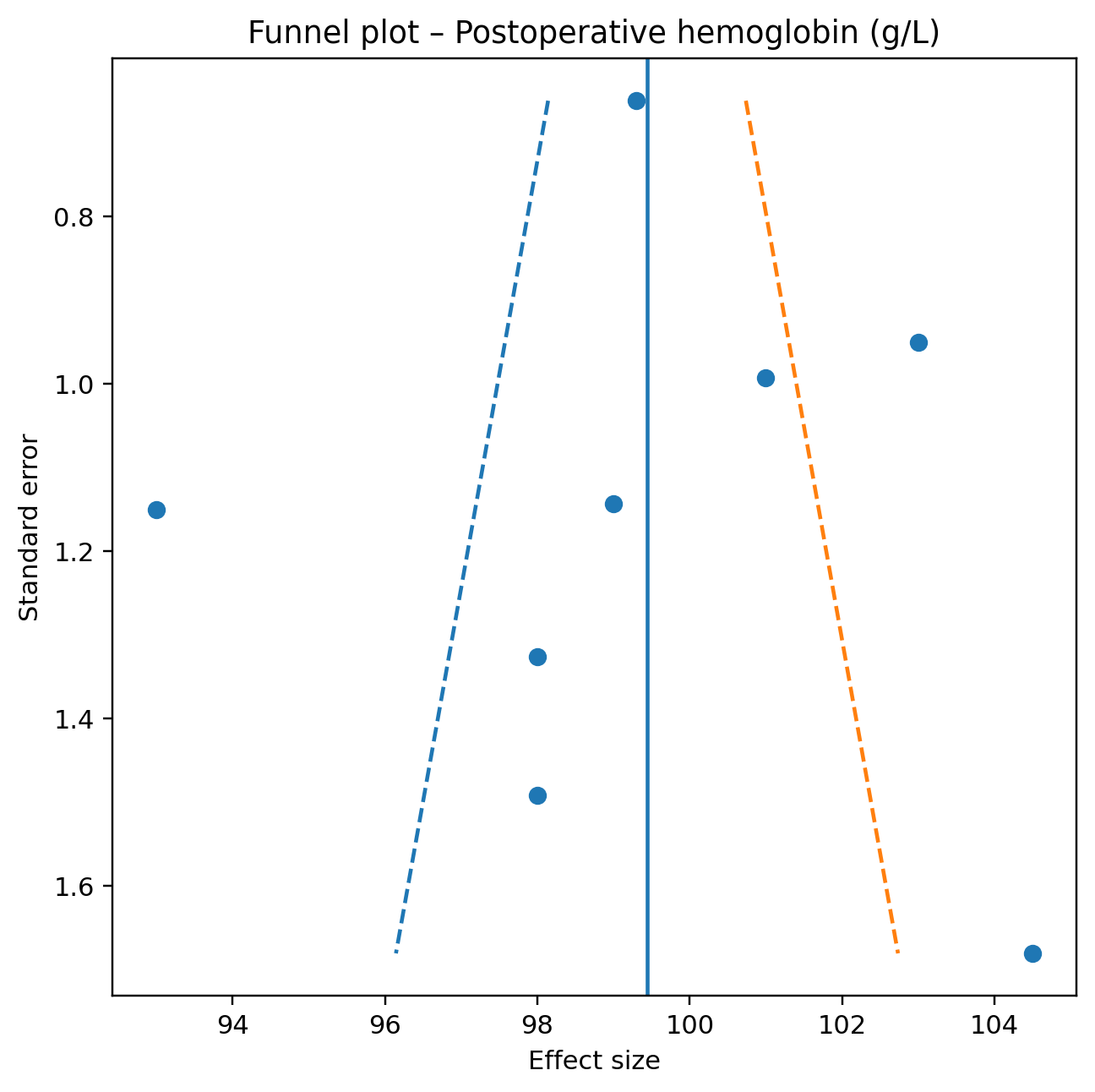

Supplement: Supplementary file 6 — Figure S6. Postoperative hemoglobin levels. Funnel plot of the studies included in the meta‐analysis of postoperative hemoglobin levels following periacetabular osteotomy (PAO). Visual inspection was performed to explore potential small‐study effects and publication bias. [file KSA-34-2576-s003.png]
